# Residential greenness and reduced depression during COVID-19: Longitudinal evidence from the Canadian Longitudinal Study on Aging

**DOI:** 10.1371/journal.pone.0329141

**Published:** 2025-08-20

**Authors:** Paul J. Villeneuve, Susanna Abraham Cottagiri, Ying Jiang, Margaret De Groh, Esme Fuller-Thomson

**Affiliations:** 1 Department of Neuroscience, Carleton University, Ottawa, Ontario, Canada; 2 Department of Public Health Sciences, Queen’s University, 62 Fifth Field Company Lane, Kingston, Ontario, Canada; 3 Applied Research Division, Center for Surveillance and Applied Research, Public Health Agency of Canada, Ottawa, Ontario, Canada; 4 Institute for Life Course & Aging, Factor Inwentash Faculty of Social Work, University of Toronto, Toronto, Ontario, Canada; 5 Factor Inwentash Faculty of Social Work, Department of Family & Community Medicine & Faculty of Nursing, University of Toronto, Toronto, Ontario, Canada; National Cheng Kung University, TAIWAN

## Abstract

**Background:**

Urban greenness has several demonstrated mental health benefits, including lower rates of depression and loneliness. Few studies have evaluated the possible benefits of greenness on depression during the COVID-19 worldwide pandemic. We investigated this topic using a prospective cohort of Canadian adults.

**Methods:**

Our study population consisted of 13,130 participants, 50 years of age and older, of the Canadian Longitudinal Study on Aging. The Center for Epidemiological Studies Depression Short Scale (CES-D-10) screening tool was used to determine whether individuals had depression at two-time points (pre-pandemic, and 6 months into the pandemic). Greenness was characterized using the maximum annual mean Normalized Difference Vegetation Index (NDVI) (500m buffer) from the pre-pandemic residential address. Logistic regression was used to estimate the odds of depression during the pandemic in relation to an interquartile range increase in the NDVI.

**Results:**

The prevalence of depression increased nearly twofold between the pre-pandemic and pandemic surveys (8.5% to 16.5% for men; 14.4% to 27.1% for women). Irrespective of depression status before the pandemic, those with higher residential greenness had lower odds of depression during the pandemic. Among those ‘not depressed’ pre-pandemic, the odds ratio (OR) of depression during the pandemic in relation to an interquartile increase in the NDVI (0.06) was 0.91 (95% CI: 0.85–0.97), while a weaker association was found for those depressed pre-pandemic (OR=0.96; 95% CI: 0.83–1.11). The inverse association between greenness and depression during the pandemic was strongest among those of lower socioeconomic status.

**Conclusions:**

Our findings suggest that green spaces in urban areas helped mitigate against depression during the pandemic.

## Introduction

Following the announcement of a global COVID-19 pandemic by the World Health Organization (WHO) in March 2020, many countries, including Canada, swiftly adopted population control measures such as social distancing to reduce disease transmission. As a result, during this time, most individuals spent the vast majority of time at home, with social interactions being restricted [1,2]. The impacts of the pandemic were multifaceted, including disruptions in healthcare services, changes in health behaviors, financial instability, and increased isolation – all of which may have exacerbated mental health issues [1]. A recent review has highlighted the profound impacts of the pandemic and its associated restrictions on the well-being of older adults, specifically loss of friendships, heightened suicidal behaviours, and increased mental health disorders [3].

The public health restrictions during the pandemic led to people spending more time at home and in their surrounding neighbourhoods. As the urban built environments exert an important influence on the health of residents, it follows that the associated impacts would likely play a greater role during the COVID-19 pandemic. An important feature of urban built environments is the availability and access to greenspaces and parks.

The beneficial health impacts of greenness on both mental and physical health have been widely studied [4–8]. Neighbourhood greenness facilitates opportunities for physical activity and social interactions [9], while providing environmental benefits such as decreases in ambient air pollution, extreme heat temperatures [10], and noise pollution [11]. Additionally, urban vegetation has been shown to reduce the risks of chronic diseases such as cardiovascular disease, stroke, mortality, and diabetes [7,12–14], which in turn positively influences mental health. Our cross-sectional analyses of participants at baseline (2011–2015) among Canadian Longitudinal Study of Aging (CLSA) participants found that residential greenness in urban areas is associated with reduced rates of depression [15], loneliness [16], and diminishes socio-economic gradients in mental health [15]. Findings from a series of epidemiological studies have similarly found that residential proximity to greenness confers mental health benefits [17–20].

As previously noted, an important consequence of the COVID-19 pandemic was that individuals spent substantially more time at home. A limited body of research has evaluated whether access to green spaces during the pandemic positively impacted mental health. For example, Lee et al found that individuals living in greener areas in the UK reported lower levels of psychological distress [21]. Similarly, in Hong Kong, proximity to greenness during a COVID-19 lockdown was associated with reduced test anxiety among university students [22]. In Nanjing City, China, residents with access to urban parks with greater amounts of greenness were found to be happier [23].

In the United States, Reid et al administered a cross-sectional survey in Denver between November 2019 and January 2021 and found that the prevalence of depression was higher among those surveyed during the pandemic compared to surveyed beforehand. Notably, they observed that improved mental health during the COVID-19 pandemic was linked to perceptions of increased green space usage and abundance [24]. Comparable findings were reported in a survey of 2060 participants in Stockholm County in Sweden [25]. Finally, a cross-sectional study by Patwary et al in Bangladesh and Egypt found those who spent time in green spaces following lockdown periods were more likely to report mental health improvements [26].

While the studies described above identified associations between increased access to greenness and mental health during the COVID-19 pandemic, they were limited by their cross-sectional designs and the lack of pre- and post-pandemic measures of depression. To our knowledge, no study has assessed changes in mental health from before to after the pandemic in relation to residential greenness. We believe a longitudinal approach offers a more robust evaluation of the benefits of greenness on mental health during the pandemic, as cross-sectional analyses are more susceptible to self-selection and participation biases. To address this research gap, we conducted an analysis of participants from the CLSA who provided data both prior to and shortly after the onset of the pandemic.

## Methods

### Study population

We constructed a cohort of individuals using data collected at two time intervals within the CLSA. The CLSA participants were between 45 and 85 years of age at the time of recruitment (between 2010 and 2015). This national stratified cohort was constructed using three sampling frames: i) Statistics Canada’s Canadian Community Health Survey – Healthy Aging; ii) recruitment from the registries of provincial health care systems; and iii) recruitment using random digit dialling of landline telephones [27,28]. The minimum age of the participants in our analysis was 50 years of age.

At baseline, the CLSA cohort was comprised of 51,338 individuals. Of those, 21,241 provided core information through telephone interviews (CLSA Tracking) and 30,097 through in-home interviews (CLSA Comprehensive). CLSA participants were surveyed at multiple points in time to provide insights into the dynamic process of aging and to capture the transitions, trajectories, and profiles of aging. The original aim of the CLSA was to contact participants every three years through 2033 [27–29]. However, to assess the impact of the COVID pandemic on physical and mental health, data collection was expanded to collect data from questionnaires (COVID-19 baseline, weekly/biweekly, monthly, and COVID exit questionnaires) administered via web or phone during April 2020 and December 2020. Only those who completed a COVID-19 baseline survey were eligible to complete the COVID-19 exit questionnaire. The first follow-up for the Comprehensive cohort was conducted from 2015–2018 and a total of 27,765 participants completed the survey, among which 18,466 of them had complete data for the two COVID cycle questionnaires.

**Fig 1:**
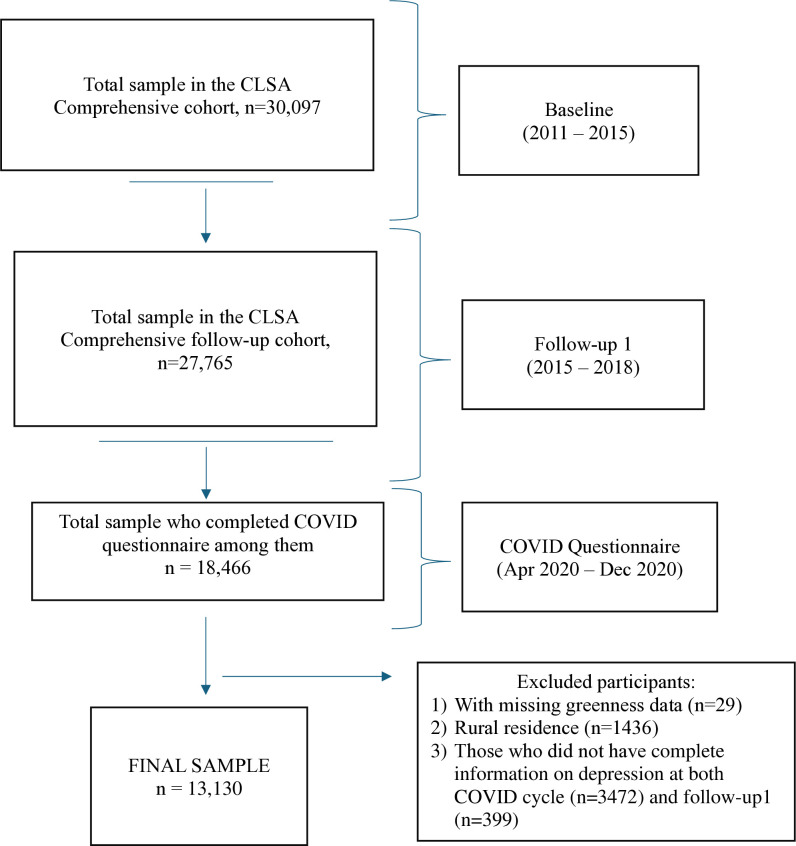
Selection of study participants.

Our analysis was restricted to the CLSA Comprehensive cohort as several of our variables of interest, such as frequency of interaction with neighbourhood, were only available for this subset. Specifically, our sample was restricted to those who completed both the initial CLSA Comprehensive Survey, the follow-up survey in 2015–2018 (n = 27,765), as well as the COVID exit questionnaire (18,466). We further restricted this subset of individuals to those living in urban areas at the time of the follow-up survey (2015–2018) because the residential postal codes used as the basis to assign greenness are not highly spatially resolved outside urban areas. Of the 18,466 individuals, a total of 13,130 urban participants had complete information on depression at both Follow-up 1 and the COVID exit cycle – this defined our final analytical sample (Fig 1).

### Characterization of residential greenness

We used the Normalized Difference Vegetation Index (NDVI), a commonly used ground-based metric of the intensity of green vegetation [30,31] to assign our residentially-based measure of greenness. The NDVI ranges from −1–1, with negative values representing water, values around zero (−0.1 to 0.1) representing bare soil or impervious surfaces, and higher positive values representing dense green vegetation. The NDVI values, indexed to DMTI Spatial Inc. postal codes, were provided by CANUE (The Canadian Urban Environmental Health Research Consortium, www.canue.ca) data repository [32,33]. A detailed description of the methodology for creating these values has been provided by CANUE [33]. In brief, CANUE extracted NDVI data from the Moderate Resolution Imaging Spectroradiometer (MODIS) that was onboard the TERRA satellites and accessed via Google Earth Engine. These data were from the MOD13Q1 V6.1 product [34,35] and provided as 16-day composites at a 250 m spatial resolution. Annual and growing season composites from the 16-day product were exported within the bounding coordinates −148 to −48 degrees longitude and 40–83 degrees latitude. These were then used to calculate annual and growing season (defined as May 1st through August 31st) metrics for all 6-character DMTI Spatial single link postal code locations in Canada, and for surrounding areas within 500 m and 1 km [34,35]. These estimates were assigned to the centroid of each participant’s six-character residential postal code based on the year of survey for baseline and follow-up 1 [31,32,36–39]. We used a pre-pandemic measure of greenness based on participants’ place of residence at the time of follow-up 1 survey that was administered between 2015 and 2018.

Following the methodological approaches of others [4,40], we restricted our sample to those with NDVI values between 0.0 and 1.0 to ensure we were isolating greenness from the potential effects of blue spaces. Additionally, we restricted analysis to urban participants as Canadian six-character postal codes are highly spatially resolved only in urban areas. We used the maximum annual mean NDVI, at a buffer of 500m as our primary measure of residential greenness.

### Ascertainment of depression

The CLSA team collected data that allowed us to classify participants according to their depression status during both the Follow-up 1 cycle (2015–2018) wave and COVID exit (May to December 2020) using the Center for Epidemiologic Studies Depression Scale short scale, also known as the CES-D-10 [41]. This measure is a 10-item Likert scale questionnaire that assesses potential depressive symptoms in the past week [42]. It has been validated across diverse population groups including both healthy and psychiatric populations [43,44] and older populations [45,46]. The summary scores for this survey ranged from 0 to 30 – with a higher cumulative score representing higher levels of depressive symptoms. Consistent with previous research [42,47–49], we adopted a threshold cut point of 10 (or higher) to classify participants as depressed for both survey cycles. We evaluated the odds of depression during COVID for those classified as depressed as well as not depressed pre-pandemic (at Follow-up 1).

### Other risk factors for depression

The follow-up 1 questionnaire included several questions on health behaviors, health status and socio-demographic factors which were considered as potential confounding or effect modifying variables in our analyses. Socio-demographic factors included biological sex, age at the time of survey completion (50–60, 61–70, 71–80 and 81+), immigration status, household size, annual household income categories (Less than $20,000, $20,000 to less than $50,000, $50,000 to less than $100,000, $100,000 to less than $150,000 and $150,000 or more) and wealth indicating the total value of savings and investments (was classified into less than $50,000, $50,000 – < $100,000, $100,000 – < $1,000,000 and $1 million or more).

Several health-related behaviours were modelled including: frequency of cigarette smoking (daily/occasionally or not at all) and alcohol consumption (4–7 times a week, 1–3 times a week, 1–3 times a month and less than once a month), physical activity levels (modelled using a composite score as high, moderate, little and none) and mobility (ability to walk: yes/no). Participants were also asked about how often they interacted with their neighbourhoods, specifically, “During the past four weeks, have you been to places in your neighbourhood, other than your own yard or apartment building?” [50]. The responses were classified into ≤ 3 and ≥4 interactions per week. Lastly, adverse experiences during the pandemic such as the death of a close person and loss of income were also considered.

### Statistical methods

First, we calculated summary statistics (mean and standard deviations) for the NDVI, at a 500m buffer, across the series of potential confounders. Frequencies are presented after rounding to the nearest base 5 unit to prevent residual disclosure of small cell counts. Cell counts that were less than 10 were suppressed. We then assessed associations between these potential confounding variables and CES-D-10 scores at the two survey dates: 1) pre-pandemic and 2) during the pandemic and could therefore determine changes in depression status during these two time periods.

We evaluated the relationship between residential greenness and depression during COVID separately for those classified pre-pandemic as “depressed” and “not depressed”. This was initially done using multivariable logistic regression models that were fit where the dependent variable was a dichotomous measure of depression and the independent variable “urban greenness” was modelled in relation to an interquartile range increase (IQR) in the maximum of annual mean NDVI at a buffer of 500m. The IQR in our study population was 0.06. We then fit multivariable logistic regression models for the same dichotomous measure of depression, but against a quartile-based classification of greenness (also based on the maximum of annual mean NDVI at a buffer of 500m).

For both exposure metrics, a series of measures of associations using incremental statistical models to better understand the potential confounding roles of specific risk factors were generated. The first set of measures of association was obtained from models that were minimally adjusted for age-group, and sex. This model was then extended to include the following variables: immigrant status, wealth, household size, mobility issues, alcohol consumption, smoking status, physical activity, death of a close person and loss of income during the pandemic. And finally, a third set of measures of association was generated that incorporated all the above variables and extended to include the frequency of interaction with neighbourhood (Table 3 and 4). The minimally adjusted model included all participants, while the further adjusted models were based on a complete case approach.

**Table 1:** Descriptive characteristics of urban participants of Canadian Longitudinal Study of Aging who completed the baseline, follow-up and COVID questionnaires (n = 13,130).

		Participants		NDVI*	
Characteristic		n^†^	(%)	Mean	Std
Sex	Male	6230	(47.5)	0.764	0.050
	Female	6900	(52.5)	0.764	0.049
Age group	50–60	2925	(22.3)	0.764	0.050
(at COVID)	61–70	4820	(36.7)	0.764	0.049
	71–80	3595	(27.4)	0.764	0.048
	81–93	1790	(13.6)	0.765	0.049
Marital Status	Married/Common-law	9155	(69.7)	0.766	0.048
(at F1)	Single	1150	(8.7)	0.754	0.055
	Separated/Divorced/Widowed	2815	(21.5)	0.762	0.050
	Missing	sup			
Household size	Single person	3220	(24.5)	0.760	0.052
(Covid)	More than one person	9650	(73.5)	0.765	0.048
	Missing	260			
Immigrant	Yes	2285	(17.4)	0.764	0.050
(Baseline)	No	10845	(82.6)	0.764	0.049
	Missing	sup			
Able to walk	Yes	12865	(98.0)	0.764	0.049
(Mobility) (at F1)	No	250	(1.9)	0.760	0.050
	Missing	15			
Smoking status	Daily/ Occasionally	730	(5.5)	0.762	0.052
(at F1)	Never	12390	(94.4)	0.764	0.049
	Missing	10			
Alcohol consumption	4–7 times a week	3675	(28.0)	0.766	0.048
(at F1)	1–3 times a week	4370	(33.3)	0.764	0.049
	1–3 times a month	2185	(16.6)	0.764	0.048
	Less than once a month	1455	(11.1)	0.761	0.051
	Missing	1450	(11.0)		
Physical activity	No activity	1015	(7.7)	0.768	0.048
(at F1)	Little activity	7870	(59.9)	0.763	0.050
	Moderate activity	3495	(26.6)	0.764	0.048
	High activity	750	(5.7)	0.768	0.047
	Missing	sup			
Neighbourhood	No interaction/ Less than once	255	(1.9)	0.776	0.046
Interactions per week	1–3 times	1160	(8.8)	0.766	0.050
(at F1)	4–6 times	2450	(18.7)	0.764	0.049
	Daily	9240	(70.4)	0.764	0.049
	Missing	25			
Household income	Less than $20,000	450	(3.4)	0.759	0.051
(at F1)	$20,000 to less than $50,000	2370	(18.1)	0.762	0.050
	$50,000 to less than $100,000	4600	(35.0)	0.764	0.049
	$100,000 to less than $150,000	2690	(20.5)	0.766	0.048
	$150,000 or more	2340	(17.8)	0.767	0.047
	Missing	680	(5.2)		
Wealth^$^	Less than $50,000	2095	(16.0)	0.766	0.048
(at F1)	$50,000 to less than $100,000	1805	(13.8)	0.763	0.050
	$100,000 to less than $1 mill	6670	(50.8)	0.765	0.049
	$1 mill or more	1510	(11.5)	0.764	0.051
	Missing	1045	(8.0)		
Death of close person	Yes	1960	(14.9)	0.764	0.047
During pandemic	No	11115	(84.7)	0.764	0.049
	Missing	55	(0.4)		
Loss of income during	Yes	1615	(12.3)	0.760	0.054
Pandemic	No	11455	(87.3)	0.765	0.048
	Missing	60	(0.4)		
	All participants	13130	(100)	0.764	0.049

F1 refers to the survey administered pre-pandemic (between 2015 and 2018)

*Based on maximum of annual mean NDVI within a 500 m circular buffer from the centroid of their residential postal code at the time of follow-up 1 interview

**Final analytical sample restricted to urban participants those who answered depression questions at both cycles (Follow-up1 and COVID-exit questionnaire)

^$^Wealth is the total value of savings and investments

^†^Frequencies were rounded to the nearest 5 to prevent residual disclosure of small cell counts

Sup = suppressed due to small cell counts.

**Table 2:** Self-reported depression ^a^ across socio demographic variables pre-pandemic and during the pandemic (n = 13,130 *).

Characteristic		Total	With depression before pandemic		With depression during pandemic		Change in prevalence
			n^†^	%	n	%	%
Sex	Male	6230	530	8.5	1025	16.5	94.1
	Female	6900	995	14.4	1865	27.1	88.2
Age group	50–60	2930	355	12.1	725	24.7	104.1
(at COVID)	61–70	4820	555	11.5	1085	22.5	95.7
	71–80	3595	375	10.4	690	19.3	85.6
	81 - 93	1790	235	13.3	395	22.0	65.4
Marital Status	Married/Common-law	9155	830	9.1	1810	19.8	117.6
(at F1)	Single	1150	200	17.2	330	28.6	66.3
	Separated/Div/Widowed	2815	490	17.4	750	26.6	52.9
	Missing	sup					
Household size	Single person	3220	590	18.4	915	28.5	54.9
(Covid)	>1 person	9650	880	9.1	1900	19.7	116.5
	Missing	260					
Immigrant	Yes	2285	245	10.6	495	21.6	103.8
(Baseline)	No	10845	1208	11.8	2395	22.1	87.3
	Missing	sup					
Able to walk	Yes	12865	1435	11.1	2785	21.6	94.6
(at F1)	No	250	85	33.9	104	41.4	22.1
	Missing	15					
Smoking status	Daily/ Occasionally	730	155	21.0	225	31.2	48.1
(at F1)	Never	12390	1365	11.0	2660	21.5	95.5
	Missing	10					
Alcohol cons.	4–7 times a week	3675	350	9.6	775	21.1	119.8
(at F1)	1–3 times a week	4370	445	10.2	915	21.0	105.9
	1–3 times a month	2180	270	12.4	490	22.4	80.6
	< 1 time a month	1455	215	14.9	360	24.6	65.1
	Missing	1450					
Physical	No activity	1015	175	17.4	255	24.9	43.1
activity (at F1)	Little activity	7870	990	12.6	1780	22.6	79.4
	Moderate activity	3495	305	8.7	710	20.3	133.3
	High activity	750	50	6.7	145	19.4	189.6
	Missing	sup					
Neighbourhood	< 1 time	255	55	21.6	70	27.6	27.8
Interactions per	1–3 times	1160	220	18.9	300	25.8	36.5
week (at F1)	4–6 times	2450	345	14.1	605	24.6	74.5
	Daily	9240	900	9.7	1910	20.7	113.4
	Missing	25					
Household	Less than $20,000	450	135	29.9	135	30.2	1.0
income (at F1)	$20,000 – < $50,000	2370	420	17.6	620	26.1	48.3
	$50,000 – < $100,000	4600	505	10.9	1010	22.0	101.8
	$100,000 – < $150,000	2690	235	8.7	540	20.1	131.0
	$150,000 or more	2340	145	6.2	410	17.4	180.6
	Missing	680					
Wealth	Less than $50,000	2095	400	19.2	580	27.6	43.8
(at F1)^$^	$50,000 – < $100,000	1805	225	12.5	440	24.5	96.0
	$100,000 – < $1,000,000	6670	645	9.7	1340	20.1	107.2
	$1 mill or more	1510	95	6.2	245	16.4	164.5
	Missing	1045					
Death of a close	Yes	1960	260	13.4	510	25.9	93.3
Person during	No	11,115	1250	11.2	2355	21.2	89.3
Pandemic	Missing	55					
Loss of income	Yes	1615	240	14.7	495	30.5	107.5
during	No	11455	1270	11.1	2370	20.7	86.5
Pandemic	Missing	55					
	All participants	13130	1520	11.6	2890	22.0	89.7
							

^a^Depression was dichotomized based on the CES-D-10. Those with a CES-D-10 score of 10 or higher were classified as depressed, those with scores less than 10 were classified as ‘not depressed’.

*Sample restricted to those who completed all questions in both the cycles

^$^Wealth is the total value of savings and investments

^†^Frequencies were rounded to the nearest 5 to prevent residual disclosure of small cell counts

Sup = suppressed due to small cell counts.

**Table 3:** Adjusted odds ratios for depression during COVID-19 by quartiles of greenness ^a^ by history of depression ^b^ among urban participants of the Canadian Longitudinal Study of Aging (n = 13,130 ^c^).

Depression status pre-pandemic (at Follow-up 1)	Quartile range (min, max)	Total n (%)	With Depression during pandemic (%)	Model 1^d^		Model 2^d^		Model 3^d^	
				OR	95% CI	OR	95% CI	OR	95% CI
Not depressed	Q1 (0.400–0.740)	3239	18.1	1.0		1.0		1.0	
(n = 11,609)	Q2 (0.750–0.770)	2854	16.0	0.86	0.75–0.98	0.82	0.70–0.96	0.82	0.70–0.96
	Q3 (0.780–0.800)	3302	16.7	0.92	0.81–1.05	0.92	0.79–1.06	0.92	0.79–1.06
	Q4 (0.810–0.880)	2214	15.9	0.85	0.73–0.98	0.81	0.68–0.96	0.81	0.68–0.96
Depressed	Q1 (0.500–0.740)	448	64.7	1.0		1.0		1.0	
(n = 1521)	Q2 (0.750–0.770)	388	62.6	0.97	0.68–1.36	0.92	0.67–1.26	0.96	0.68–1.35
	Q3 (0.780–0.800)	407	60.4	0.88	0.63–1.22	0.88	0.65–1.19	0.87	0.62–1.21
	Q4 (0.810–0.870)	278	59.4	0.80	0.55–1.15	0.78	0.55–1.09	0.80	0.55–1.15

^a^Based on maximum of annual mean NDVI within a 500 m circular buffer from the centroid of their residential postal code at the time of interview

^b^As reported at Follow-up1. Those with a CES-D-10 score of 10 or higher were classified as depressed

^c^Only urban participants included as greenness is spatially resolved in these areas

^d^Confounders based on p-value, relevance in the literature and collinearity

Model 1: Adjusted for age and sex.

Model 2: Adjusted for age, sex, immigrant status, wealth, household size, mobility issues, alcohol consumption, smoking status, physical activity, death of close person and loss of income during the pandemic

Model 3: Adjusted for age, sex, immigrant status, wealth, household size, mobility issues, alcohol consumption, smoking status, physical activity, death of close person and loss of income during the pandemic and frequency of interaction with neighbourhood

Model 1 was based on all participants, while models 2 and 3 were based on a complete case approach

**Table 4:** Adjusted odds ratios for depression during the COVID-19 pandemic in relation to an interquartile range (IQR = 0.06) increase in greenness^a^ based on depression status pre-pandemic^b^ among urban participants of the Canadian Longitudinal Study of Aging (n = 13,130 ^c^).

Depression status pre-pandemic	Depression during COVID	Participants n (%)	Model 1		Model 2		Model 3^d^	
			OR	95% CI	OR	95% CI	OR	95% CI
Not Depressed	No	9662 (83.2%)	1.0		1.0		1.0	
(n = 11609)	Yes	1947 (16.8%)	0.91	0.85–0.96	0.91	0.85–0.97	0.91	0.85–0.97
Depressed	No	577 (37.9%)	1.0		1.0		1.0	
(n = 1521)	Yes	944 (62.1%)	0.93	0.82–1.05	0.97	0.83–1.11	0.96	0.83–1.11

^a^Based on maximum of annual mean NDVI within a 500 m circular buffer from the centroid of their residential postal code at the time of interview

^b^As reported at the Follow-up1 survey administered between 2015 and 2018. Those with a CES-D-10 score of 10 or higher were classified as depressed

^c^Only urban participants included as greenness is spatially resolved in these areas

^d^Confounders based on p-value, relevance in the literature and collinearity

Model 1: Adjusted for age and sex

Model 2: Adjusted for age, sex, immigrant status, wealth, household size, mobility issues, alcohol consumption, smoking status, physical activity, death of close person and loss of income during the pandemic

Model 3: Adjusted for age, sex, immigrant status, wealth, household size, mobility issues, alcohol consumption, smoking status, physical activity, death of close person and loss of income during the pandemic and frequency of interaction with neighbourhood

Model 1 was based on all participants, while models 2 and 3 were based on a complete case approach

**Fig 2:**
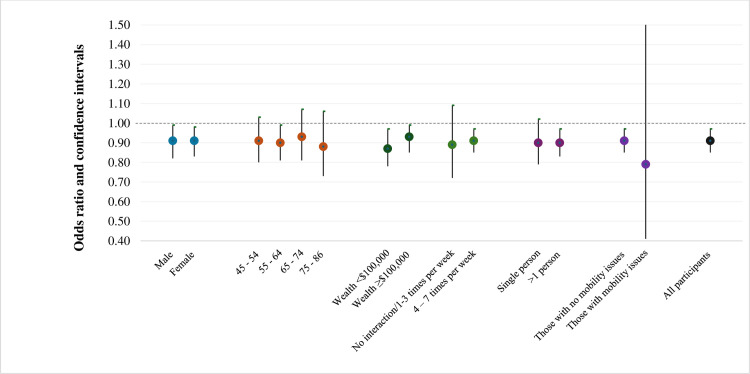
Adjusted odds ratios for depression during COVID-19 in relation to an interquartile range (IQR = 0.06) increase in greenness ^a^ before selected effect modifiers for those ‘not depressed’ pre-pandemic among urban participants of the Canadian Longitudinal Study of Aging ^c^ (n = 11,609). a Based on maximum of annual mean NDVI within a 500 m circular buffer from the centroid of their residential postal code at the time of interview. b As reported at Follow-up1. c Models adjusted for age, sex, immigrant status, wealth, household size, mobility issues, alcohol consumption, smoking status, physical activity, death of close person and loss of income during the pandemic, frequency of interaction with neighbourhood.

**Fig 3:**
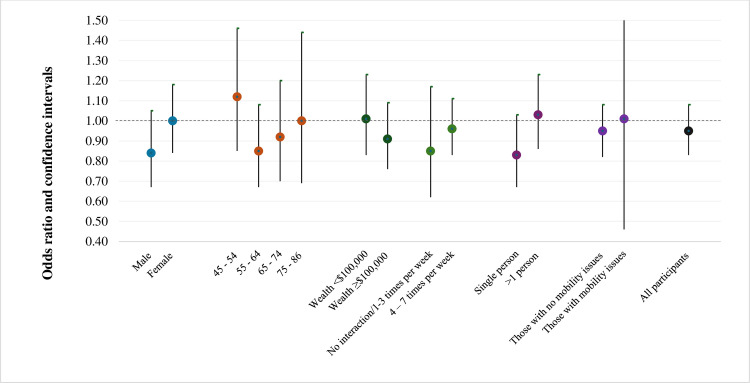
Adjusted odds ratios for depression during COVID-19 in relation to an interquartile range (IQR = 0.06) increase in greenness ^a^ by key effect modifiers for those classified as depressed pre-pandemic ^b^ among urban participants of the Canadian Longitudinal Study of Aging ^c^ (n = 1521). a Based on maximum of annual mean NDVI within a 500 m circular buffer from the centroid of their residential postal code at the time of interview. b As reported at Follow-up1. c Models adjusted for age, sex, immigrant status, wealth, household size, mobility issues, alcohol consumption, smoking status, physical activity, death of close person and loss of income during the pandemic, frequency of interaction with neighbourhood.

**Fig 4:**
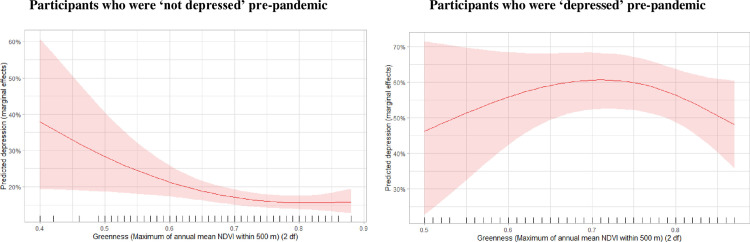
Marginal effects* of residential proximity to greenness on depression status during the pandemic according to depression status measured pre-pandemic. * The marginal effects were adjusted for age, sex, immigrant status, wealth, household size, mobility issues, alcohol consumption, smoking status, physical activity, death of close person and loss of income during the pandemic, frequency of interaction with neighbourhood.

Stratified analyses were conducted across the sexes, four age-groups, categories of wealth household size, frequency of interaction with neighbourhoods, and mobility status – to explore if these factors modified associations between mental health and greenness during the pandemic. For variables (wealth and interactions with neighbourhood) with strata sample sizes that were low we recoded similar strata appropriately to improve model stability (Fig 2 and 3). Lastly, we generated an exposure-response plot to illustrate the association between urban residential greenness and the occurrence of incident depression for the two groups (Fig 4).

All multivariate regression modelling was done using Stata version 18 (StataCorp LLC, College Station, TX, USA).

### Ethics approval

The study protocol of the CLSA was approved by 13 Research Ethics Boards (REB) across Canada associated with each CLSA collection site. This analysis of CLSA data was approved by Carleton University’s REB (Clearance #: 120725).

## Results

Our study population consisted of 13,130 individuals, and of these, 52.5% were women (Table 1). The participants ranged in age from 50 through 93 years at the time of completion of the second survey (i.e., during the COVID pandemic). The mean NDVI did not vary substantially across the categories of many descriptive characteristics. However, those with higher household income tended to live in greener areas, as did those who were married, and those who lived in a multi-person households (Table 1).

The overall prevalence of depression (assessed by the CES-D-10 scale) was nearly double that from before the pandemic (22.0% versus 11.6%). The prevalence of depression was highest in single person households (28.5%) and those with lower household incomes (Table 2).

Of the 13,130 respondents, 11.6% (n = 1,521) were classified as ‘depressed’ pre-pandemic. There was an inverse exposure-response relationship between residential greenness and depression during the pandemic for those who were ‘not depressed’ prior to the pandemic. Specifically, the corresponding adjusted odds ratio among individuals who lived in areas within the upper quartile of residential greenness (500 m buffer) was 0.81 (95% CI: 0.68–0.96) when compared to those in the lowest quartile. A similar but not statistically significant association was found among individuals who were depressed pre-pandemic as the corresponding odds ratio was 0.80 (95% CI: 0.55–1.15) (Table 3). When we modelled NDVI as a continuous variable, we found a statistically significant and slightly stronger inverse association with depression during the pandemic for those classified as ‘not depressed’ pre-pandemic. This adjusted odds ratio in relation to an interquartile range increase in the NDVI (500 m buffer) was 0.91 (95% CI: 0.85–0.97) among those with no history of depression (Table 4).

We also conducted various stratified analyses to assess effect modification by age, sex, socio-economic status and health behaviours and these findings are presented in Figs 2 and 3.

For those classified as ‘not depressed’ before the pandemic, we found an inverse association between greenness and CES-D-10 scores across both sexes, and most age-groups. Notably, the beneficial associations of greenness and depression (during the pandemic) was more pronounced in the lower wealth group than higher wealth group. We also observed inverse associations between greenness and CES-D-10 scores during the pandemic among those who interacted less with their neighbourhoods compared to those who interacted more. Lastly, we also found that the beneficial associations of greenness on depression during the pandemic was stronger for those with mobility issues when compared to those who did not (Fig 2).

## Discussion

Our analysis of longitudinal data from the CLSA suggests that residential proximity to greenness in urban areas reduced the risk of depression during the early phases of the COVID-19 pandemic. Spline analyses indicated that these benefits were stronger among those who were not depressed before the pandemic. Additionally, the protective effects of greenness were stronger for those with lower wealth, while no substantive differences were observed between women and men.

Several studies have reported poorer mental health outcomes during the COVID-19 pandemic among individuals with pre-existing mental health conditions compared to those without. For example, a recent UK study found that people with a history of anxiety, depression, post-traumatic stress disorder, or eating disorders were more likely to report worsened mental health than their counterparts [51]. Similar results emerged from a multi-country study spanning Bosnia and Herzegovina, Canada, France, Germany, Iran, Italy, Pakistan, Poland, Spain, Switzerland, Turkey, and the United States, where over half of the patients experienced a worsening of their pre-existing psychiatric conditions [52]. Our finding that residential greenness was protective against depression during the pandemic—but only reached statistical significance among those not classified as depressed pre-pandemic—contributes further knowledge to this topic.

A key finding was that residential greenness was seemingly more effective at reducing depression among those of lower wealth than higher wealth. However, this pattern was only observed among those who did not have pre-existing depression. Pre-pandemic, the increased beneficial effects of greenness among those of lower socio-economic status has been reported in several North American and European studies [4,53,54], including our previous cross-sectional studies [4,15]. For low wealth groups, the protective effects during the pandemic could be because green spaces act as a refuge from financial and other stressors and the restorative and therapeutic effects of accessing nature.

We also found that residential greenness was more protective against depression for those with mobility issues compared to those who did not have mobility issues but only among those who did not have pre-existing depression. This could be because of the crucial role that residential and immediate green spaces played when other nature related public spaces such as parks where inaccessible or restricted. The protective effect restricted to only those without pre-existing depression as mentioned above could be due to additional health care and social barriers for the subgroup [55,56].

While our study reports some benefits of greenness on depression during the pandemic, it is important to recognize that some physical distancing measures during the COVID-19 pandemic in Canada led to the closure of public parks and recreational centers in some jurisdictions at various times. This policy differed from those of other countries, such as Norway, where an increased use of green spaces was noted during the pandemic [57]. As we see it, the Canadian policies related to restricting public park use has two important implications for the interpretation of our findings. First, it would have been preferable to have greenness measures that capture to access and availability to proximal green spaces rather than the overall measure of vegetation that we modelled. The second implication is that the benefits that we observed could have been ever greater had these park restrictions not been in place.

We observed a stronger protective effect for those who interacted less with their neighborhoods. It is important to note that the frequency of interaction with neighborhoods was measured pre-pandemic, and therefore, some misclassification of these behaviours during the pandemic is inevitable. We do note that during lockdowns and restrictions in Canada, nature walks and outdoor exercise were encouraged as long as individuals adhered to public health guidelines [58]. It is possible that individuals who reported interacting more with their neighbourhoods prior to the pandemic modified their social routines and limit their time-activity patterns to align with public health restrictions. Pre-pandemic, increased protective effects for those who interacted more with their neighborhoods were reported by cross-sectional studies [5] including our study [15]. However, we found no literature on this during the pandemic. Our findings are suggestive of the beneficial effect of greenness depend on the frequency as well as prevailing restrictions imposed.

Key strengths of our study include i) a large number of participants from across many urban Canadian areas and ii) the longitudinal nature of outcome (depression), which allowed us to isolate the effects of greenness on depression for those with and without pre-existing. Additionally, our study was able to assess greenness and depression association during the pandemic across sociodemographic groups in a Canadian context. We were also able to assess associations by effect modifiers often not available in literature such as by how often individuals interacted with their neighbourhoods.

There are several limitations to be acknowledged. Although our study population is comprised of a large number of individuals, it is predominantly Caucasian and of higher socioeconomic status when compared to the general population. Additionally, as the COVID surveys were administered online or by phone it is likely that elderly and individuals who do not use internet or the phone regularly who could be at high risk for mental health issues were less likely to have participated. Importantly, our study population is comprised of those who live in urban areas. We further acknowledge that the CLSA excluded those who were in long-term care. Thus, our sample excludes some of the most vulnerable older Canadians, and for all the reasons listed above, one should be cautious in generalizing these findings to Canadians as a whole.

With respect to the characterization of greenness, we were only able to use a cross-sectional measure at Follow-up1 (right before the pandemic). Although it is unlikely that a substantive number of participants moved between this date and the start of the pandemic, – we were unable to directly assess this quantitatively. Moreover, like other epidemiological studies based on the NDVI, we were not able to evaluate the influence that specific features of greenness (i.e., access, the type of vegetation, the biodiversity, etc.) may have exerted. Neighbourhood-based measure of greenness using a postal code may be imperfect as it does not capture interactions with nature that may occur at more distal locations. While our risk estimates were adjusted for other risk factors, residual confounding may occur due to biases in using self-reported data, as well as inability to track time-dependent changes in these factors.

Our measure of mental health was based on the CES-D-10, and we acknowledge that this survey instrument is a screening and not a diagnostic tool. It is a shortened version of the CES-D-20 instrument [59] and therefore may miss less commonly experienced symptoms, and this could result in reduced ability to identify those with milder depression. Despite this limitation, in our study population CES-D-10 has the advantage of being administered at multiple times, thereby allowing for inferences to be made based on within-individual changes. Moreover, it is also worth noting that the CES-10 short form has been demonstrated to have high reliability and internal consistency [60], as well as validity in identifying those with depressive symptoms [61].

Overall, our study suggests that residential greenness in urbanites has mental health benefits during challenging times like the pandemic, especially for those without pre-existing depression. The substantial deterioration in CES-D-10 scores in our study population underlines the need for mental health supports during the pandemic. We also found neighbourhood greenness had larger beneficial effects for vulnerable groups, such as those in lower income groups and those with mobility disorders.
