# Correction: Residential greenness and reduced depression during COVID-19: Longitudinal evidence from the Canadian Longitudinal Study on Aging

**DOI:** 10.1371/journal.pone.0361587

**Published:** 2026-10-27

**Authors:** Paul J. Villeneuve, Susanna Abraham Cottagiri, Ying Jiang, Margaret De Groh, Esme Fuller-Thomson

The Acknowledgements and Disclaimer sections were omitted from the published article. The missing sections should read as follows:
